# A Retrospective Analysis of the Effect of Anlotinib in Patients With Lung Cancer With or Without Previous Antiangiogenic Therapy

**DOI:** 10.3389/fonc.2021.788837

**Published:** 2021-12-23

**Authors:** Jiaojiao Suo, Yu Sun, Yan Fu, Weigang Xiu, Xuanwei Zhang, Yan Wang, Jiang Zhu

**Affiliations:** ^1^ Department of Thoracic Oncology, West China Hospital, Sichuan University, Chengdu, China; ^2^ Radiation Physics Center, West China Hospital, Sichuan University, Chengdu, China; ^3^ Reproductive Medical Center, Department of Obstetrics and Gynecology, West China 2nd University Hospital, Sichuan University, Chengdu, China; ^4^ Key laboratory of Birth Defects and Related Diseases of Women and Children (Sichuan University), Ministry of Education, Chengdu, China

**Keywords:** lung cancer, antiangiogenic, efficacy, survival, anlotinib

## Abstract

**Objective:**

The purpose of this study was to initially investigate the effect of previous antiangiogenic therapy (bevacizumab and endostatin) on the efficacy of anlotinib in patients with advanced or metastatic lung cancer (LC).

**Methods:**

We retrospectively collected the clinical data of patients with LC treated with anlotinib and divided them into group A (treated with anlotinib after the failure of previous antiangiogenic drugs and group B (no prior use of antiangiogenic drugs). We used propensity score matching (PSM) for confounding factors between the groups. Progression-free survival (PFS) and overall survival (OS) were also recorded.

**Results:**

A total of 160 patients were included in the analysis. The median OS in groups A and group B was 11.8 months and 16.1 months (P=0.120), whereas the median PFS was 3.1 months and 4.7 months (P=0.009), respectively. Moreover, the objective response rate (ORR) of the two groups was 9.6% and 10.4% (P=0.874), and the disease control rate (DCR) was 71.1% and 80.5% (P=0.165).

After PSM (n=46), baseline characteristics were comparable between groups A and B. Furthermore, the median OS of the two groups was 14.6 months and 16.2 months (P=0.320), whereas the median PFS was 3.5 months and 4.5 months (P=0.040), respectively. Moreover, the ORR of the two groups were 13.0% and 10.9% (P=0.748), and the DCR were 78.3% and 82.6% (P=0.599), respectively.

**Conclusions:**

Previous antiangiogenic treatments may affect the PFS of patients who receive anlotinib later, but it might not affect the patient’s ORR and OS.

## Introduction

In recent years, malignant tumors have become a major global public health problem, among which lung cancer (LC) is one of the most common malignant tumors (1). In 2020, the newly diagnosed patients with LC accounted for 11.4% of all malignant tumors, and 1.8 million people died of LC, which posed a great burden to individuals, families, and society (2).

The NCCN guidelines recommend first- and second-line treatment for patients with LC; however, there are fewer options for third- and further- line treatments (3). More patients with LC remain in good physical condition after receiving the recommended standard treatment, and they need safe and more effective third- and further-line treatments. Among these few treatment options is anlotinib (4–7).

Currently, a significant number of patients were treated with bevacizumab or endostatin antiangiogenic therapy prior to anlotinib (8, 9). A retrospective study of 118 patients with advanced LC demonstrated that there is no benefit from continued second-line bevacizumab treatment after progression of first-line treatment with bevacizumab or endostatin (10). After the failure of single-target bevacizumab therapy, the effectiveness of switching to another small-molecule multi-target antiangiogenic agent deserves further investigation. Retrospective studies have shown that previous antiangiogenic therapies (bevacizumab or endostatin) may not affect the efficacy of retro-line anlotinib therapy, suggesting that there may be no cross-resistance between anlotinib and other antiangiogenic agents (11, 12).The possible mechanism is that anlotinib not only inhibits the VEGF pathway, but also inhibited other angiogenic bypasses. In LC, due to the small sample size of existing studies, it is unclear whether antiangiogenic drugs can be cross-line used to induce sustained vascular inhibition. Therefore, the present study retrospectively analyzed the efficacy of anlotinib in patients with advanced LC, and preliminarily observed whether previous antiangiogenic drugs affected anlotinib efficacy.

## Materials and Methods

### Patients

We scanned the medical data of patients with LC treated with anlotinib who were admitted at the West China Hospital from June 2018 to January 2021. Patients treated with anlotinib after the failure of previous antiangiogenic therapy (bevacizumab or endostatin), were assigned to group A (83 patients). By contrast, patients treated with anlotinib without any previous antiangiogenic therapy, were assigned to group B (77 patients).

### Therapeutic Regimen

The initial dose of anlotinib was 12mg or 10mg orally once daily on days 1-14, every 3 weeks. The dose for some patients was reduced to 10mg/day or 8mg/day when they became intolerant to adverse reactions. Anlotinib was continued until disease progression or when patients remained intolerant to adverse reactions. The anlotinib regimens include:(1)anlotinib monotherapy,(2)anlotinib combined with chemotherapy (no restriction on chemotherapy regimen), (3) anlotinib combined with immune checkpoint inhibitors (anti-PD-1/L1 antibodies), (4) anlotinib combined with targeted agents (including epidermal growth factor receptor TKI and ALK/ROS inhibitors), and (5) anlotinib combined with local radiotherapy (no limitation on the radiotherapy site and dose).

### Efficacy Evaluation

Radiographic examinations were performed to evaluate the efficacy two cycles after initiation, and then every two cycles or periodically according to clinical conditions. Efficacy evaluation was directly performed by the researchers using the Picture Archiving and Communication Systems in the hospital according to the Response Evaluation Criteria In Solid Tumor 1.1, with reference to imaging reports and clinical practice. The best response evaluation was the best response record from the beginning to the end of anlotinib treatment. Progression-free survival (PFS) was defined as the time from anlotinib initiation to the presence of objective evidence of disease progression (or death for any reason). Overall survival (OS) was defined as the time from anlotinib initiation until death, or loss to follow-up or reaching the study observation deadline. Adverse events were documented according to the electronic medical records of our hospital, and the incidence of adverse events was lower than the actual incidence.

### Statistical Analysis

Statistical analyses were performed using R version 4.0.5. Categorical variables were compared using Pearson’s chi-square test or Fisher’s exact test. Survival curves were created using the Kaplan–Meier method. The log-rank test was used for univariate analysis of PFS and OS. Propensity score matching (PSM) was performed using 1:1 nearest neighbor matching (caliper 0.2). The matching factors were sex, age (< 60 years old, ≥ 60 years old), histological subtype (adenocarcinoma, squamous cell carcinoma, others), clinical-stage (stage IV), and the number of previous treatment lines (<3 lines, ≥3 lines). Statistical significance was set at P<0.05 (both sides).

## Results

### Baseline Patient Characteristics

**Figure 1:**
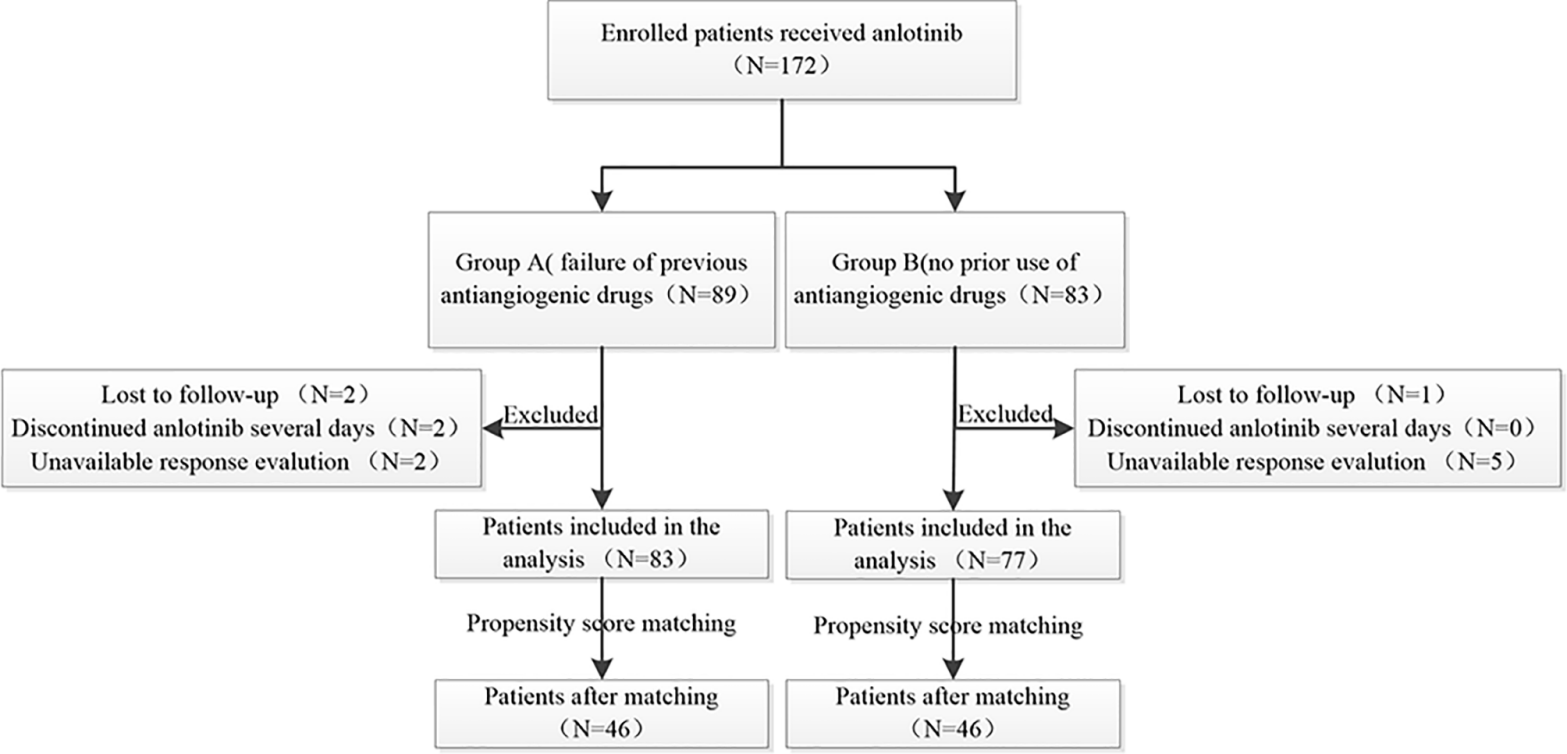
The research flow chart.

A total of 172 patients were treated with anlotinib. Three patients were lost to follow-up, two cases were discontinued anlotinib after several days, one of which was due to dizziness, and the other was due to fatigue, hypertension, and proteinuria. Efficacy evaluation could not be performed in seven patients due to imaging inaccessibility, therefore they were excluded. A total of 160 patients were included in the analysis, 83 of whom had received prior antiangiogenic therapy (bevacizumab or endostatin) (group A) and 77 of whom did not receive antiangiogenic therapy (group B).The research flowchart is shown in **Figure 1**, and the baseline patient characteristics are shown in **Table 1**. As seen in the table, the two patients groups were not balanced in terms of sex (P=0.020), age (P=0.000), pathological type (P=0.000), clinical-stage (P=0.015), PD-L1 expression level (P=0.019), smoking history (P=0.02), number of previous treatment lines (P=0.000), history of targeted therapy (P=0.024), and therapeutic schemes (P=0.014) (**Table 1**). PSM was used to control the influence of confounding factors between the two groups. After matching, the baseline characteristics of the two groups became comparable (**Table 2**).

**Table 1:** Baseline characteristics of patients.

Characteristics	Group A (n = 83,%)	Group B (n = 77,%)	P value
**Gender**			0.020*
Male	41 (49.4)	52 (67.5)	
Female	42 (50.6)	25 (32.5)	
**Age (years)**			0.000*
median (range)	55 (34-80)	63 (29-85)	
<60	59 (71.1)	33 (42.9)	
≥60	24 (28.9)	44 (57.1)	
**Pathology**			0.000*
Adenocarcinoma	71 (85.5)	42 (54.5)	
Squamous cell carcinoma	6 (7.2)	19 (24.7)	
Others	6 (7.2)	16 (20.8)	
**Stage**			0.015*
IIIB/IIIC	0 (0)	7 (9.1)	
IV	83 (100.0)	70 (90.9)	
**PD-L1**			0.019*
0	42 (50.6)	21 (27.3)	
1-49	12 (14.5)	13 (16.9)	
<50	11 (13.3)	13 (16.9)	
NA	18 (21.7)	30 (39.0)	
**Driver Gene status**			0.037*
Yes	34 (41.0)	17 (22.1)	
None	26 (31.3)	33 (42.9)	
Unknown	23 (27.7)	27 (31.3)	
**Brain metastases**			0.150
Yes	35 (42.2)	24 (31.2)	
No	48 (57.8)	53 (68.8)	
**Liver metastasis**			0.654
Yes	18 (21.7)	19 (24.7)	
No	65 (78.3)	58 (75.3)	
**ECOG**			0.895
0	10 (12.0)	9 (11.7)	
1	37 (44.6)	33 (42.9)	
2	32 (38.6)	29 (37.7)	
3	4 (4.8)	6 (7.8)	
**Smoking history**			0.02*
Yes	23 (27.7)	35 (45.5)	
No	60 (72.3)	42 (54.5)	
**No. of previous therapy lines**			0.000*
**median (range)**	3	2	
<3	36 (43.4)	57 (74.0)	
≥3	47 (56.6)	20 (26.0)	
**History of targeted therapy**			0.024*
Yes	32 (38.6)	17 (22.1)	
No	51 (61.4)	60 (77.9)	
**Previous antiangiogenic drugs**			–
Bevacizumab	73 (88.0)	0 (0)	
Endostar	10 (12.0)	0 (0)	
**Therapeutic schemes**			0.014*
Anlotinib monotherapy	47 (56.6)	40 (51.9)	
Chemotherapy+anlotinib	23 (27.7)	11 (14.3)	
Immunotherapy+anlotinib	6 (7.2)	17 (22.1)	
Radiotherapy+anlotinib	4 (4.8)	8 (10.4)	
Targeted therapy+anlotinib	3 (3.6)	1 (1.3)	

NA, not available; ECOG, Eastern Cooperative Oncology Group; *indicates that the difference was statistically significant.

**Table 2:** Comparison of baseline characteristics of patients after 1:1 matching of propensity score matching.

Characteristics	Group A (n = 46,%)	Group B (n = 46,%)	P value
**Gender**			0.529
Male	24 (52.2)	27 (58.7)	
Female	22 (47.8%)	19 (41.3)	
**Age (years)**			0.833
median (range)	57 (34-80)	57 (29-82)	
<60	26 (56.5)	27 (58.7)	
≥60	20 (43.5)	19 (41.3)	
**Pathology**			0.778
Adenocarcinoma	34 (73.9)	31 (67.4)	
Squamous cell carcinoma	6 (13.0)	7 (15.2)	
Others	6 (13.0)	8 (17.4)	
**Stage**			1.000
IIIB/IIIC	0 (0)	0 (0)	
IV	46 (100.0)	46 (100.0)	
**PD-L1**			0.239
0	21 (45.7)	16 (34.8)	
1-49	8 (17.4)	4 (8.7)	
>50	6 (13.0)	7 (15.2)	
NA	11 (23.9)	19 (41.3)	
**Driver Gene status**			0.652
Yes	17 (37.0)	14 (30.4)	
None	13 (28.3)	17 (37.0)	
Unknown	16 (34.8)	15 (32.6)	
**Brain metastases**			0.832
Yes	19 (41.3)	18 (39.1)	
No	27 (58.7)	28 (60.9)	
**Liver metastasis**			0.470
Yes	10 (21.7)	13 (28.3)	
No	36 (78.3)	33 (71.7)	
**ECOG**			0.393
0	6 (13.0)	7 (15.2)	
1	24 (52.2)	18 (39.1)	
2	14 (30.4)	17 (37.0)	
3	2 (4.3)	4 (8.7)	
**Smoking history**			0.825
Yes	31 (67.4)	30 (65.2)	
No	15 (32.6)	16 (34.8)	
**No. of previous therapy lines**			0.400
<3	24 (52.2)	28 (60.9)	
≥3	22 (47.8)	18 (39.1)	
**History of targeted therapy**			1.000
Yes	15 (32.6)	15 (32.6)	
No	31 (67.4)	31 (67.4)	
**Therapeutic schemes**			0.053
Anlotinib monotherapy	19 (41.3)	24 (52.2)	
Chemotherapy+anlotinib	15 (32.6)	8 (17.4)	
Immunotherapy+anlotinib	5 (10.9)	11 (23.9)	
Radiotherapy+anlotinib	4 (8.7)	3 (6.5)	
Targeted therapy+anlotinib	3 (6.5)	0 (0)	

NA, not available; ECOG, Eastern Cooperative Oncology Group.

### Comparison of Efficacy Between the Two Groups

The best response in group A was partial response (PR) (n =8), stable disease (SD) (n = 51) and progressive disease (PD) (n =24). In group B, the best response PR (n =8), SD (n = 55) and PD (n =14). The objective response rate (ORR) and disease control rate (DCR) of the two groups were 9.6% in group A vs. 10.4% in group B (P=0.874), and 71.1% in group A vs. 80.5% in group B (P=0.165), respectively (**Table 3**).

**Table 3:** Comparison of the best response between the two Groups.

Best response	Before matching		P value	After matching		P value
	Group A (n = 83)	Group B (n = 77)		Group A (n = 46)	Group B (n = 46)	
**PR**	8	8	–	6	5	–
**SD**	51	55	–	30	33	–
**PD**	24	14	–	10	8	–
**ORR**	9.6%	10.4%	0.874	13.0%	10.9%	0.748
**DCR**	71.1%	80.5%	0.165	78.3%	82.6%	0.599

PR, partial response; SD, stable disease; PD, progression disease; ORR, objective response rate; DCR, disease control rate.

**Figure 2:**
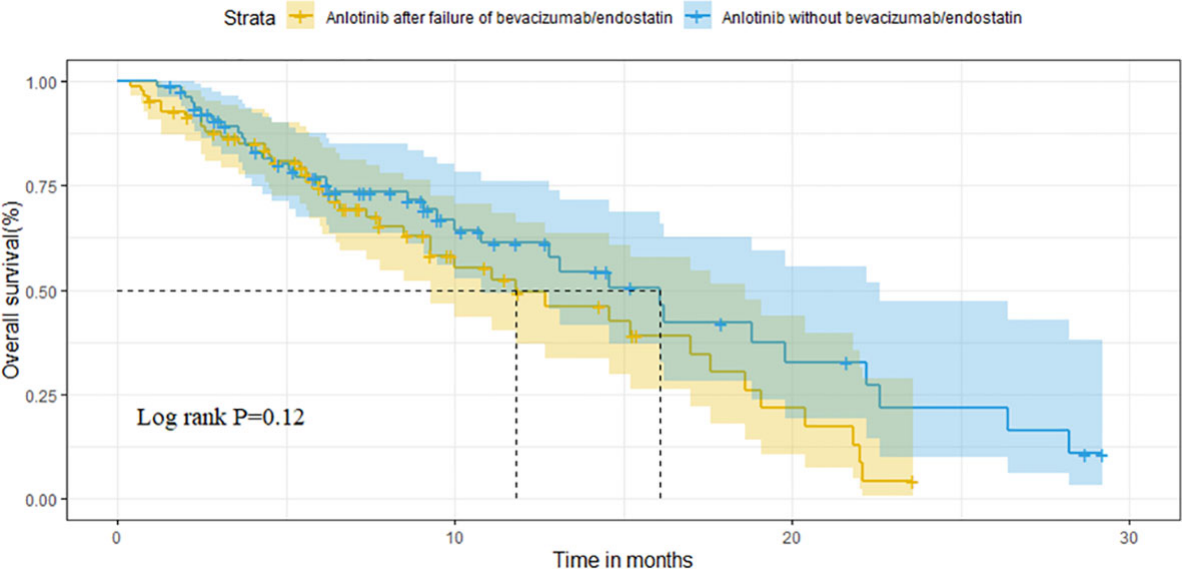
Comparison of OS between the two groups.

**Figure 3:**
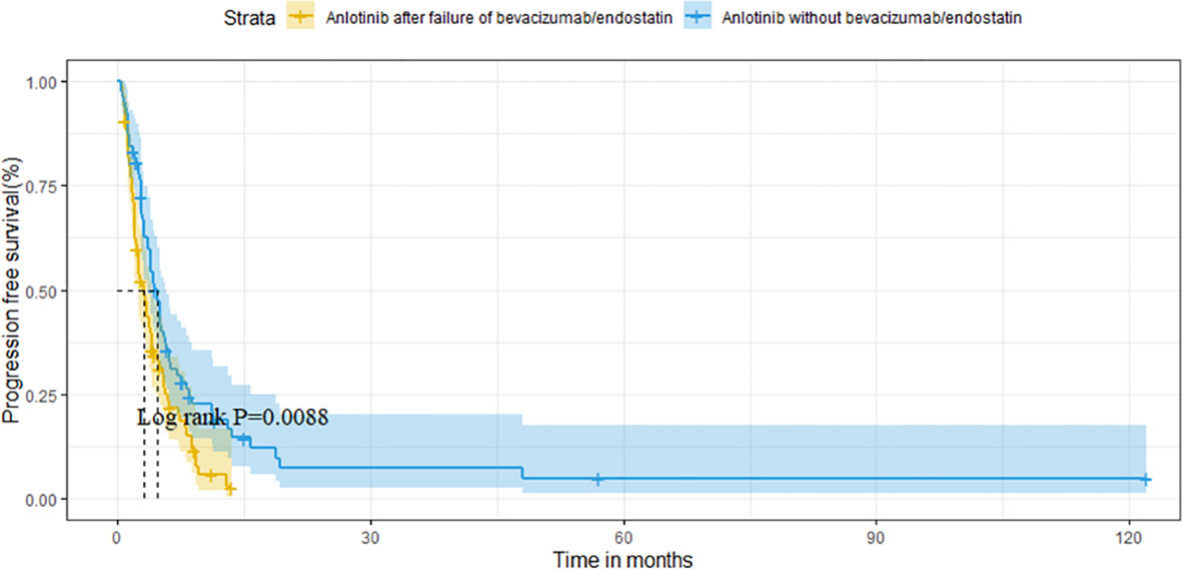
Comparison of PFS between the two groups.

For the overall population, the median OS and PFS were was 14.6 months (95%CI 11.1-18.1) and 3.8 months (95%CI 3.1-4.5), respectively. The median OS was 11.8 months (95%CI 6.7-16.9) in group A vs. 16.1 months (95%CI 12.6-19.6) in group B (P=0.121) (**Figure 2**). Median PFS was 3.1 months vs. 4.7 months (95%CI 3.9-5.5),(P=0.009; **Figure 3**).

**Figure 4:**
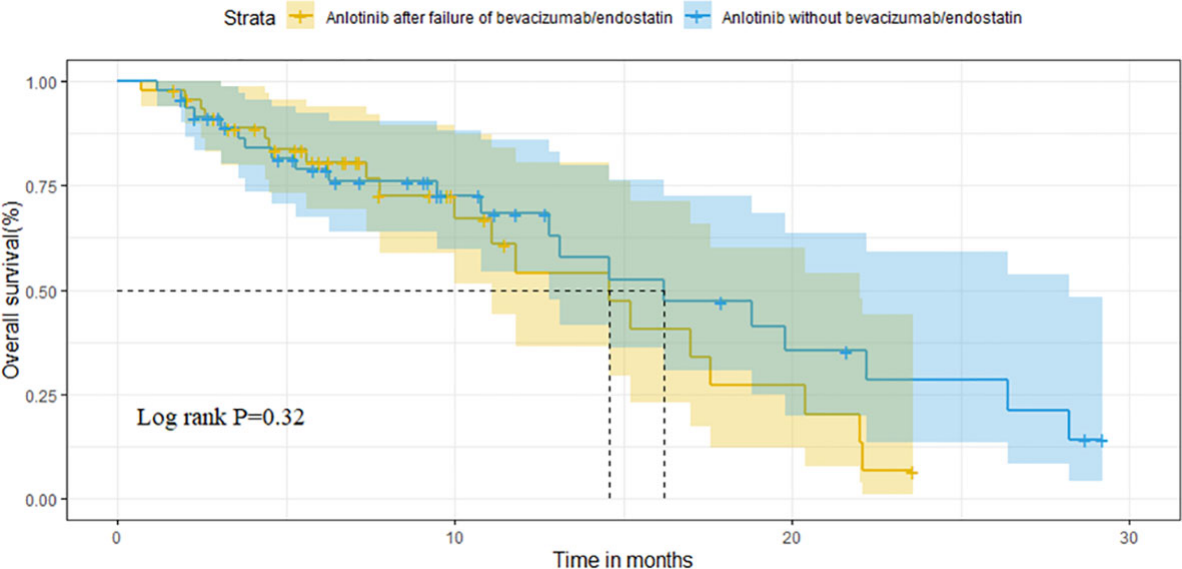
Comparison of OS between the two groups after matching.

**Figure 5:**
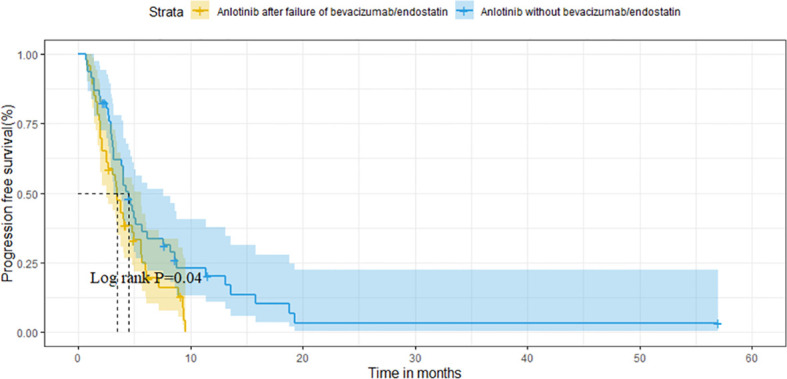
Comparison of PFS between the two groups after matching.

After matching, the ORR of the two groups was 13.0% vs. 10.9% (P=0.748). The DCR of two groups was 78.3% vs. 82.6% (P=0.599), respectively (**Table 3**). The median OS of the two groups was 14.6 months (95%CI 10.1-19.1) in group A vs. 16.2 months (95%CI 9.2-23.2) in group B (P=0.320; **Figure 4**). Median PFS was 3.5 months (95%CI 2.7-4.3) and 4.5 months (95%CI 3.7-5.3) (P=0.040; **Figure 5**).

### Analysis of the Subsequent Treatment After Anlotinib Progression

After anlotinib progression, 57 patients (68.7%) in group A and 61 patients (79.2%) in group B received subsequent therapy. However, there was no statistically significant difference in subsequent line therapy between the two groups before and after matching. The most common posterior line treatments in both groups were targeted therapy, immunotherapy, and radiotherapy, whereas the other relatively uncommon treatments included chemotherapy and liver interventional therapy. However, data regarding the posterior line treatment of a few patients were unavailable (**Table 4**).

**Table 4:** Subsequent treatment after the progression of anlotinib therapy.

**Subsequent treatment**	**Before matching**		**P value**	**After matching**		**P value**
	**Group A (n=83)**	**Group B (n=77)**		**Group A (n=46)**	**Group B (n=46)**	
**None**	26(31.3)	16(20.8)	0.130	10(21.7)	8(17.4)	0.599
**Targeted therapy**	20(24.1)	13(16.9)	0.260	12(26.1)	12(26.1)	1.000
**Immunotherapy**	17(20.5)	16(20.8)	0.963	9(19.6)	12(26.1)	0.456
**Radiotherapy**	13(15.7)	9(11.7)	0.466	11(23.9)	6(13.0)	0.179
**Chemotherapy**	8(9.6)	14(18.2)	0.117	4(8.7)	8(17.4)	0.216
**Interventioal therapy**	0(0)	1(1.3)	0.481	0(0)	0(0)	1.000
**Unknown**	6(7.2)	6(7.8)	0.892	5(10.9)	3(6.5)	0.711

### Effect of Previous Antiangiogenic Therapy on Anlotinib

The effect of previous antiangiogenic treatment on anlotinib in group A was further analyzed, the Kaplan-Meier analysis showed that the PFS for anlotinib in the bevacizumab and endostatin groups were 3.2 and 2.1 months, respectively (P =0.973). The PFS for anlotinib in the PR, SD, and PD groups that received prior antiangiogenic therapy were 2.7 months, 3.1 months and 3.8 months (P =0.918), respectively. Similarly, after grouping according to the PFS achieved from previous antiangiogenic treatment, the PFS for anlotinib in the shorter (≤6 months) and longer (>6 months) groups were 2.9 and 3.3 months (P=0.592), respectively. The OS data for the anlotinib group after the progression of previous antiangiogenic therapy are detailed in **Supplementary Table 1**.

### Univariate and Multivariate Analyses of Anlotinib Clinical Factors

The univariate analysis showed that female patients (P=0.021), patients with ECOG scores of 0-2 (P=0.001), and patients with brain metastases (P=0.015) had longer PFS (**Table 5**). Factors with P<0.05 in the univariate analysis and those clinically associated with prognostic factors (liver metastasis) were included in the multivariate analysis. ECOG score (P=0.002), brain metastasis (P=0.006), and liver metastasis (P=0.006) were factors found to independently influence PFS (**Table 6**). In terms of OS, the univariate analysis showed that patients with an ECOG score of 0-2 had significantly longer OS than those with an ECOG score of 3, whereas other factors did not affect the OS of the patients (**Table 5**).

**Table 5:** Univariate analysis of PFS and OS (COX regression analysis).

	PFS		OS		
Characteristics	HR (95% CI)	P value	HR (95% CI)	P value
**Gender** (Female vs male)	0.661 (0.465-0.939)	0.021*	1.468 (0.918-2.349)	0.109
**Age** (≥60 years vs<60 years)	1.055 (0.747-1.491)	0.760	0.993 (0.628-1.569)	0.975
**ECOG** (3 vs 0-2)	5.807 (2.107-16.006)	0.001*	18.203 (5.209-63.612)	0.000*
**Brain metastasis** (Yes vs No)	0.886 (0.804-0.977)	0.015*	0.926 (0.814-1.053)	0.238
**Liver metastasis** (Yes vs No)	1.451 (0.993-2.121)	0.054	1.339 (0.829-2.164)	0.232
**Smoking history **(Yes vs No)	1.044 (0.732-1.489)	0.813	1.330 (0.826-2.140)	0.241
				
**No. of previous therapy lines** (≥3 vs <3)	1.122 (0.797-1.579)	0.510	0.957 (0.606-1.512)	0.852
				
**History of targeted therapy** (Yes vs No)	1.386 (0.969-1.983)	0.074	1.503 (0.946-2.388)	0.084

*indicates that the difference was statistically significant.

**Table 6:** Multivariate analysis of PFS (COX regression analysis).

Characteristics	HR (95%CI)	P value
**ECOG** (3 vs 0-2)	5.092 (1.818-14.261)	0.002*
**Brain metastases** (Yes vs No)	0.865 (0.780-0.960)	0.006*
**Liver metastasis** (Yes vs No)	1.756 (1.178-2.618)	0.006*
**Gender** (Female vs male)	–	0.134

*indicates that the difference was statistically significant.

### Safety Analysis

No life-threatening adverse events associated with anlotinib were documented in this study. However, six patients were spontaneously discontinued in group A due to being intolerant to adverse reactions, including one patient who experienced vaginal bleeding and fatigue, one who experienced hematuria, three who experienced skin symptoms, and one who experienced nausea and vomiting. In group B, four patients were discontinued due to adverse reaction intolerance, including one who experienced nausea and vomiting, one who had hand and foot syndrome, one who had an acute stroke, and one who had upper gastrointestinal bleeding after an esophageal cancer diagnosis.

There was no statistically significant difference in the incidence of adverse events between the two groups. The most common adverse events in group A were skin symptoms (24.1%), including rash, pruritus, hand and foot syndrome, followed by hypertension (8.4%) and fatigue (6.0%). The other relatively rare adverse events of concern included vaginal bleeding (3.6%), nosebleeds (2.4%), and hematuria (1.2%). In group B, the most common adverse events were skin symptoms (14.3%), hypertension (10.4%), and anorexia (10.4%), followed by diarrhea (7.8%) and fatigue (7.8%). The other relatively uncommon adverse events of concern included hemoptysis (2.6%), upper gastrointestinal bleeding (1.3%), and stroke (1.3%) (**Table 7**).

**Table 7:** Adverse events with anlotinib.

Adverse events	Group A (n = 83,%)	Group B (n = 77,%)	P value
**General reaction**			
Fatigue	5 (6.0)	6 (7.8)	0.659
Decreased appetite	4 (4.8)	8 (10.4)	0.181
Weight loss	1 (1.2)	0 (0)	1.000
**Gastrointestinal reactions**			
Diarrhea	2 (2.4)	6 (7.8)	0.231
Oral mucositis	3 (3.6)	3 (3.9)	1.000
Nausea/vomiting	3 (3.6)	3 (5.2)	1.000
Constipation	0 (0)	1 (1.3)	0.481
Hiccup	1 (1.2)	1 (1.3)	1.000
**Cardiovascular symptoms**			
Hypertension	7 (8.4)	8 (10.4)	0.672
Premature atrial contractions	1 (1.2)	0 (0)	1.000
**Skin symptom**	20 (24.1)	11 (14.3)	0.117
**Proteinuria**	1 (1.2)	0 (0)	1.000
**Hemorrhage**			
Vaginal hemorrhage	3 (3.6)	0 (0)	0.271
Nasal hemorrhage	2 (2.4)	0 (0)	0.498
Hematuria	1 (1.2)	0 (0)	1.000
Upper gastrointestinal hemorrhage	0 (0)	1 (1.3)	0.481
Hemoptysis	0 (0)	2 (2.6)	0.230
**Myelosuppression**	3 (3.6)	3 (3.9)	1.000
**Neurologic symptom**			
Dizziness	1 (1.2)	1 (1.3)	1.000
Insomnia	1 (1.2)	0 (0)	1.000
Stroke	0 (0)	1 (1.3)	0.481
**Hypothyroidism**	0 (0)	1 (1.3)	0.481

## Discussion

Angiogenesis plays an important role in tumor growth, proliferation, and metastasis of tumors (13). Excluding nintedanib (14) and anlotinib (5, 7), most small-molecule TKIs, such as sorafenib, sunitinib and apatinib, can significantly prolong the PFS of patients, but do not significantly increase the OS (13, 15–19). By contrast, anlotinib has become one of the few multi-target angiogenesis inhibitors with survival benefits (5). In the ALTER0303 trial, the vast majority of patients had not previously received antiangiogenic drugs; therefore, it is worth studying whether similar survival benefits can be achieved with anlotinib in these patients after progression of antiangiogenic therapy. The present study therefore aimed to answer the question of whether prior use of antiangiogenic therapy influences anlotinib efficacy and whether the efficacy of previous antiangiogenic therapy affects the efficacy of anlotinib antiangiogenic therapy.

In this study, the median PFS of the entire cohort was 3.8 months, whereas the median OS was 14.6 months, which also confirmed that in the real world, anlotinib could bring PFS and OS benefits among patients with advanced LC; furthermore, and the benefit seemed not inferior to that of previously reported second-line docetaxel and immune checkpoint inhibitors (20, 21). In addition, the PFS in our study was similar to that reported in the ALTER0303 trial, with a slightly longer OS than that in ALTER0303, which might be due to the higher subsequent-line treatment rate in our study.

We also found that patients who had previously received bevacizumab and endostatin had a shorter PFS (3.1 months vs. 4.7 months); however, the ORR, DCR, and OS were similar to those who had received the first antiangiogenic therapy (anlotinib). Considering that the baseline characteristics and subsequent treatment imbalance between our two patient groups may lead to results bias, we used PSM to reduce intergroup confounders. After PSM, we also found that previous antiangiogenic therapy only affected PFS in patients treated with anlotinib, but could not affect OS. Failure of previous antiangiogenic therapy may be accompanied by resistance to VEGF pathways and the emergence of new resistance mechanisms, which may be related to the shorter PFS of some patients receiving anlotinib therapy; moreover, this survival benefit might be due to the multi-target inhibition of anlotinib (22).

Furthermore, it is of interest whether the efficacy of previous antiangiogenic therapy predicts anlotinib efficacy. We found that previous drug therapy (bevacizumab or endostatin), the best response (PR, SD or PD) of the prior antiangiogenic therapy, and PFS (≤6 months or > 6 months) related to prior antiangiogenic therapy all have no significant effect on the efficacy (PFS and OS) among patients receiving anlotinib (P>0.05). These results suggest that anlotinib efficacy may be independent of the previous antiangiogenic therapy and the sensitivity of previous antiangiogenic therapy. Notably, our multivariate analysis found that patients with brain metastasis had longer PFS than those with other metastatic sites (without brain metastasis), hence we hypothesized that a small -molecule agent (anlotinib) could enter the blood-brain barrier to function.

The incidence of adverse events recorded in both of our patient groups was low and similar to that reported in the literature (23–26). In this study, the proportion of patients that required rechecking of ECG, thyroid function and urine routine was small. Therefore, the incidence of abnormal ECG, increased thyroid-stimulating hormone (TSH) and proteinuria was lower than the actual situation and the incidence reported in previous studies. Therefore, it is necessary to further strengthen the monitoring of adverse reactions during treatment.

In this study, the most common subsequent therapy after anlotinib failure was targeted therapy and immunotherapy. With the popularity of more targeted agents and immunotherapy drugs, as well as their lower toxicity compared to chemoradiotherapy, targeted therapy and immunotherapy have become the alternatives for patients with poor ECOG scores after multiline therapy. This may be one reason why the median OS in our study was slightly longer than that in the ALTER0303 trail.

### Limitations

Our study has several limitations. The results of this study were based on the experience of a single-center and a small sample size. The baseline characteristics of the patients in the two groups were influenced by confounding factors before matching. After PSM, the number of patients available for analysis was halved, which reduced the representativeness of our cohort, and could also have introduced certain limitations in the results. The variety of drugs used in combination with anlotinib might have also affected the outcomes.

## Conclusions

Based on the limited data available in this study, anlotinib may bring PFS and OS benefits to patients with advanced LC. Previous antiangiogenic treatments may affect the PFS of patients who receive anlotinib later, but this might not affect the patient’s ORR and OS. Therefore, the use of anlotinib in patients with advanced LC after the progression of antiangiogenic therapy is worthy of subsequent prospective, large-sample clinical studies.

## Data Availability Statement

The raw data supporting the conclusions of this article will be made available by the authors, without undue reservation.

## Ethics Statement 

The studies involving human participants were reviewed and approved by Biomedical Research Ethics Committee, West China Hospital, Sichuan University(approval number: 2020162). The ethics committee waived the requirement of written informed consent for participation.

## Author Contributions

All authors contributed to the study conception and design. JS, YS, and YF: Data collection, analysis and drafting. WX and XZ: Data analysis and essay structure and revision of manuscripts. YW: Determination of topics, essay structure and revision of manuscripts. JZ: In charge of the whole study, perform the final revision of the manuscript. All authors commented on previous versions of the manuscript. All authors contributed to the article and approved the submitted version.

## Conflict of Interest

The authors declare that the research was conducted in the absence of any commercial or financial relationships that could be construed as a potential conflict of interest.

## Publisher’s Note

All claims expressed in this article are solely those of the authors and do not necessarily represent those of their affiliated organizations, or those of the publisher, the editors and the reviewers. Any product that may be evaluated in this article, or claim that may be made by its manufacturer, is not guaranteed or endorsed by the publisher.
