# Supplementary material for: A Retrospective Analysis of the Effect of Anlotinib in Patients With Lung Cancer With or Without Previous Antiangiogenic Therapy
Source: Front Oncol. 2021 Dec 23;11:788837. doi: 10.3389/fonc.2021.788837 (PMC8732369; doi:10.3389/fonc.2021.788837)
Supplement: Supplementary file 1 [file Table_1.docx]

| Table 1 Analysis of anlotinib efficacy after the failure of antiangiogenic therapy | | | | |
| --- | --- | --- | --- | --- |
|  | PFS（months） | P value | OS（months） | P value |
| **Previous antiangiogenic drugs** |  | 0.973 |  | 0.249 |
| Bevacizumab (n=73) | 3.2（2.3-4.1） |  | 11.8（8.3-15.3） |  |
| Endostar (n=10) | 2.1（0.0-4.9） |  | 15.2（6.1-24.3） |  |
| **Optimal response of Previous antiangiogenic therapy** |  | 0.918 |  | 0.859 |
| PR(n=21) | 2.7(0.9-4.5) |  | 17.0（5.6-28.4） |  |
| SD(n=42) | 3.1(2.0-4.2) |  | 10.0（4.4-15.6） |  |
| PD(n=20) | 3.8(1.7-5.9) |  | 15.2（5.2-25.2） |  |
| **PFS of previous antiangiogenic therapy** |  | 0.592 |  | 0.131 |
| ≤6months(n=47) | 2.9（1.7-4.1） |  | 11.1（6.6-15.6） |  |
| ＞6months(n=36) | 3.3（1.8-4.8） |  | 12.7（3.1-22.3） |  |
